# “Teen Fled Danger into the Arms of Death”: The Political Agenda Setting Effect of Australian News Media Framing of Violence Against Women

**DOI:** 10.1177/10778012241228291

**Published:** 2024-01-31

**Authors:** Catherine Son, Victoria Fielding

**Affiliations:** 1 The University of Adelaide, Adelaide, SA, Australia

**Keywords:** violence against women, domestic violence, media framing, political agenda setting

## Abstract

News framing of violence against women (VAW) has important implications for public understanding of this epidemic problem in Australian society, and in turn, politicians’ impetus to act. This article uses a frame-building model to analyze media reporting of three cases of VAW. The murder of Eurydice Dixon, who was killed by a stranger, was framed thematically and received substantial media attention. Conversely, Larissa Beilby and Qi Yu, who were killed in incidents of domestic violence (DV), were framed episodically and received less coverage. The impact of this differential media attention is compared to public and political responses to theorize that thematic frames create a larger political agenda-setting effect, despite DV presenting a larger societal problem than stranger violence.

## Introduction

On June 13, 2018, Australians were shocked to learn of the death of Eurydice Dixon, a young woman who was murdered by a stranger in a park in Melbourne, Victoria, while walking home late at night. She had been violently assaulted, and her body was found by a passerby the following morning. Eurydice's murder attracted substantial news media coverage about the broader cultural and societal problem in Australia of violence against women—henceforth referred to as VAW. In response, prominent media commentators urged the need to address VAW at a societal level. Others argued that Eurydice's murder reflected a broader law and order problem in Melbourne. Following Eurydice's murder, politicians from all three levels of government in Australia, including the Premier of Victoria, Daniel Andrews, and Australian Prime Minister Malcolm Turnbull, expressed condolences and sympathy for Eurydice's death.

Within a 3-week period of Eurydice's murder, two other women were also violently killed by men in Australia—Larissa Beilby and Qi Yu. Larissa was killed by her partner, and her remains were found in a barrel several days later. Subsequently in this study, incidences of women being killed by a male partner will be referred to as domestic violence (DV). Qi, on the other hand, was killed by her male flatmate, reportedly due to a dispute over money. While there is no indication that Qi was romantically involved with her killer, her death is included in this study as an example of DV for two reasons. Firstly, Qi lived with her killer, which situates her murder along similar lines to family or DV. Additionally, neither woman's death generated anywhere near the media or public attention afforded to Eurydice's murder. Unlike Eurydice, there were no statements from high-profile public figures and politicians mourning Qi and Larissa's deaths.

Statistically, DV is a far more common occurrence than violent acts perpetrated by strangers. In 2022, the Australian, state and territory governments released “The National Plan to End Violence against Women and Children 2022–2032,” which was described as addressing a “problem of epidemic proportions in Australia” (DHS, 2022). In 2021/22, 35% of women over 15 had experienced violence from someone known to them, and 11% by a stranger (ABS, 2023). In 2019/2020, a woman was killed in Australia by an intimate partner on average every 10 days (Serpell et al., 2022).

Australian society's understanding of the problem of VAW, and the prevalence of DV in proportion to stranger violence, is predominantly communicated via mainstream news media. Scholars regularly use the theoretical field of framing, and the related theory of agenda-setting, to explore the influence of media reporting on topics such as VAW, on public opinion, discourse, political agendas and political action (Entman, 2007; Sellers et al., 2014). Despite the media's powerful influence on political outcomes, there is a large body of research which shows that rather than framing VAW as a social and cultural problem related to entrenched gendered roles and power imbalance (Morgan & Politoff, 2012), news reporting about VAW “frequently mirrors society's confusion and ambivalence” about this issue (Sutherland et al., 2016, p. 34). Where there is apathy on an issue among the public, politicians are unlikely to be called on to act. News reporting about VAW thus impacts the public's understanding of VAW as a societal problem, and political willingness to address it.

This research extends knowledge of how the news media build frames about incidents of VAW and DV by interrogating news discourses that frame such incidents either episodically as a personal/individual responsibility, or thematically as a societal responsibility (Iyengar, 1990). Furthermore, this research contributes new knowledge about the framing of VAW by examining whether framing such incidents thematically or episodically influences a political agenda-setting effect. To date, few studies have specifically examined how frames are built when reporting victims who are murdered by a stranger, in contrast to the framing of DV victims. Using Entman et al.'s (2009) “diachronic framing process model,” this study focuses on the reciprocal relationship and feedback loop between VAW news coverage and the political agenda to address VAW. It does this by exploring the types of frames that sustain the length of time that VAW incidents remain in the news and in turn, are prominent on the public and political agenda.

## Literature Review

### Episodic and Thematic Framing

Research indicates that media framing of an issue bears a strong correlation with public perceptions of the matter. Entman (1993) has described framing as emphasizing certain aspects of a topic or issue to make them particularly meaningful to news consumers, while downplaying or overlooking other aspects of a story. Framing also influences social understanding of an issue through the repetition of news discourses that are intended to emphasize a particular idea or outcome (Entman et al., 2009). Framing in news discourse can be categorized as episodic or thematic. “Episodic” framing presents a specific instance of a topic or issue in a news report with little contextual information (Iyengar, 1990). In contrast, “thematic” framing contextualizes an individual story in terms of a broader topic or issue (Iyengar, 1990).

Framing a story episodically or thematically also apportions blame for the issue, either individually or to broader cultural and systemic factors (Iyengar, 1990). Episodic and thematic framing also influences public opinion toward an issue. Thematic stories are framed as being more significant to the community, due to their contextualization as reflective of a pressing issue faced by the community (Shah, 2019). Consequently, media framing influences social and political attitudes toward important issues and can serve to “define the terms of a debate” (Tankard, 2001, p. 97).

### Framing of News Discourses About VAW

Although the most common form of VAW is DV and the majority of sexual assaults are perpetrated by a person known to the victim (Heise, 2018), most news reports about VAW overlook these facts and instead favor more sensationalized stories about women who are attacked by an unknown perpetrator. In the United States, news reports about VAW focus on the most exceptional or sensational cases, which are often perpetrated by strangers in public places (Carter, 1998; Meyers, 1997). It is a similar story in Australian news reporting, where until recently, all but the most sensational cases of DV were largely ignored by the Australian media (Hawley et al., 2017).

Focusing on reporting exceptional or sensational stories of attacks by strangers overlooks the widespread societal and cultural problem of VAW more broadly, and DV in particular. By only reporting “exceptional” random acts of violence, the most common forms of VAW such as DV are framed as the norm and are therefore often considered by the media to be less newsworthy (Berns, 2017; Carter, 1998; Meyers, 1997). Perpetrators of DV are also framed in news discourse as “different” or “abnormal,” which implies that certain “types” of people are more likely to commit such acts (Bullock & Cubert, 2002; Worthington, 2013). Such framing further individualizes the problem by creating an assumption that these are rare, episodic crimes perpetrated by “crazy” or “disturbed” individuals, and do not reflect a widespread cultural or societal problem.

The news media exerts a powerful influence over public perceptions and social attitudes toward violent crimes, and in particular, VAW (Fairbairn & Dawson, 2013). When a story about a violent attack is framed thematically as part of a widespread problem of VAW, pressure increases on politicians and the authorities to respond to the incident (Meyers, 1997, p. 53). Although news discourses predominantly center on cases of VAW where the perpetrator is a stranger, studies examining news reporting of VAW largely focus on DV and homicide. Many studies show that news stories about DV generally employ episodic framing, rather than framing the issue thematically as part of a societal problem of VAW (Berns, 1999; Bullock & Cubert, 2002; Carlyle et al., 2008; Hawley et al., 2017; Sellers et al., 2014). For instance, Sutherland et al.'s study of over 4,500 VAW news reports found 61% of stories were episodic, 21% thematic, and 18% a mixture of both (2019, p. 4). Easteal et al. (2019, p. 458) describe how their study of Australian media reporting of two cases of intimate homicide found these crimes are framed as “simultaneously random and specific, dramatic and mundane: not a societal problem, not a gendered problem, but a misfortune that befalls problematic individuals.”

Consequently, episodic news framing of DV as an individual concern shifts responsibility toward the individuals involved, rather than pressuring politicians and broader society to remedy the problem (Hawley et al., 2017; Meyers, 1997). Moreover, by focusing on individual aspects of DV cases, instead of thematically framing the issue as representative of a broader societal problem, deeper public debate of the causes and solutions of VAW is overlooked (Fairbairn & Dawson, 2013).

Further emphasizing the role of individual responsibility in DV incidents is the prevalence of news reports framing victims as somehow contributory to the crimes committed against them (Berns, 1999; Saroca, 2013). For example, studies show that questioning a victim's decision to stay in a relationship with their abuser is a key frame in media reporting of DV (Berns, 1999; Fairbairn & Dawson, 2013). Other victim-blaming frames applied in news discourses about DV include focusing on aspects of the victims’ lives, such as substance abuse, financial situation, or social isolation, to frame them as “abnormal” and thus somehow contributory to the violence perpetrated against them (Bullock & Cubert, 2002; Fairbairn & Dawson, 2013; Meyers, 1997). Victim-blaming in news framing of DV stories is also more prevalent than for women who were attacked by a stranger because the victim–perpetrator relationship implies “the victim should have known better than to be involved with their abuser” (Fairbairn & Dawson, 2013, p. 152). By extension, such individualistic framing establishes low public and media expectations of politicians to respond to these incidents.

### Frame Building About VAW

Where most studies about media framing of VAW or DV focus on the frames found in media content, this study explores factors which influence why journalists use thematic or episodic frames in these contexts. Although framing theory is widely used to understand the makeup of news content, less is known about the impacts on the production and distribution of these frames (Borah, 2011, p. 250; D'Angelo, 2018, p. 233; Hänggli, 2020).

Responding to the need to better understand the production of frames, scholars are increasingly interested in frame building, which is the process by which frames are constructed and communicated by journalists (Bartholomé et al., 2015; Boesman et al., 2017; Dekavalla, 2016). Within this sub-area of framing research, there are very few studies focused on how journalists build frames about VAW. There are however studies about the practice of reporting about VAW which provide useful insights into frame building. For example, Easteal et al. (2022) analyzed whether Australian journalists trained to report VAW had improved their practices. This study found there was some evidence of improvement, such as the inclusion of more societal context. Another study relevant to frame building is Simons and Morgan's (2018) analysis of how journalists’ sourcing practices are changing the way VAW is reported in Australia. Their study focused on three Australian news outlets, two of which had made concerted efforts to report VAW as a social or thematic problem (Simons & Morgan, 2018). They found that although police remained an important source for journalists writing about VAW, since police in Australia are working to improve reporting in this area, journalists’ continued reliance on police sources positively impacted the quality and volume of stories (Simons & Morgan, 2018).

On the same topic, Morgan and Simons (2018) used interviews with journalists and editors to describe how individual news producers were influenced to evolve their reporting from episodic to thematic when particular cases of VAW resonated with them. The key characteristics of these resonating cases centered on the interviewee perceiving them as “perfectly innocent” or a “good victim” (Morgan & Simons, 2018). One case mentioned was Jill Meagher, another woman who was killed by a stranger while walking home in Melbourne in 2012. Jill was described by interviewees as an “innocent target of ‘stranger danger’,” whose death caused an outpouring of community grief, which the interviewees took as a sign that the public wanted to talk about VAW (Morgan & Simons, 2018, p. 1172). The other case was the murder of Sargun Raji, who was killed by her former partner in 2012. Morgan and Simons describe how this case helped the news producers see VAW as a systemic problem, particularly because Raji “followed all the rules” by taking out an intervention order, and “didn’t get a gun and shoot him” (Morgan & Simons, 2018, p. 1171). These findings suggest that thematic reporting is influenced by how journalists view a victim of VAW or DV, particularly their perception of how innocent a murdered woman is in the context of the crime.

The reasons why journalists, as above, tend to frame VAW episodically, focus on crimes committed by strangers, and imply victims hold some responsibility for what happened to them, are complex and varied. Berns (2017) suggests a key reason for this framing is that emphasis on violence committed by male strangers against women can be presented as a crime or medical problem, rather than a systemic and gendered social problem, reflecting underlying sexism and attitudes which are difficult to address. A study of frame building in relation to VAW, Shah's (2019) study of Indian reporting of the 2012 Delhi gang-rape, suggests audiences are calling for the media to recognize VAW as a social rather than an individual problem. Shah (2019) found reporting moved from episodic when the crime first occurred, to thematic over time. Shah suggests this shift in reporting from episodic to thematic was influenced by audience responses on social media, with activists and other members of the public calling on the media to focus more on societal causes of violence against women (Shah, 2019)—demonstrating a feedback loop between the public and media agenda.

To compare journalist frame building in reporting about the murders of Eurydice Dixon, Larissa Beilby, and Qi Yu, in this research, Entman et al.'s (2009, p. 178) diachronic framing process model is used. This model helps to explain the complex influences on frame building and to account for the way frames cascade and evolve from the framing junctures of culture, elite debate, the media, and the public. The model assumes that as frames evolve through these frame junctures, the frames most used will dominate the next level's interpretation of an issue, which will eventually come to dominate the media audience's interpretation (Entman et al., 2009).

In this study, the key framing junctures analyzed in relation to VAW reporting are news frames and the public opinion indicators of community responses as well as elite strategic frames among politicians across three levels of the Australian government. This model theorizes that news frames influence public opinion indicators, including the frames used by strategic framers such as politicians and nonstrategic framers such as members of the public (Entman et al., 2009). Entman et al. (2009) suggest that these strategic politicians’ frames will subsequently influence media network framing back up the chain, shaping the media's evolving framing response. In discussing their model, Entman et al. (2009, p. 188) suggest more research is needed to understand how the production and circulation of frames is influenced by “the feedback loops that trace the flow of political power among competing media, competing elites, and mass publics.” This article responds to this call by comparing thematic and episodic framing of VAW and politicians’ strategic responses to this media framing.

### Media Reporting and Political Agenda-Setting

Numerous studies outside of the field of frame building help to demonstrate a link between issues featured prominently on the news media agenda, and issues that are prioritized by politicians (Dearing & Rogers, 1996; Van der Pas et al., 2017; Vliegenthart et al., 2016; Walgrave & Van Aelst, 2016). This close relationship is established by the news media alerting politicians through news reports to issues that the public will likely expect them to respond to (Sevenans, 2017). Politicians thus perceive high levels of media attention toward an issue as indicative of public interest and opinions about the matter (Sevenans, 2017; Walgrave & Van Aelst, 2006; Yanovitzky, 2002), which in turn creates “a sense of urgency among policy makers to generate immediate, short-term solutions to public problems” (Yanovitzky, 2002, pp. 444–445).

There is contestation, however, about whether the salience of an issue on the media agenda results in meaningful, long-term political action such as policy change (Elmelund-Praestekaer & Wien, 2008; Sevenans, 2017; Walgrave & Van Aelst, 2006; Yanovitzky, 2002). For instance, Elmelund-Praestekaer and Wien (2008) found media hype creates political discussion and puts issues on the political agenda, but doesn’t necessarily result in meaningful changes to policy or regulations—changes they concede usually need a much longer time frame to carry out.

Nonetheless, the prominence and salience of an issue on the media agenda significantly boosts the likelihood of attention and responses from politicians to the matter. These responses have been found to generally represent short-term symbolic actions, such as statements in parliament, rather than substantive long-term policy reforms (Elmelund-Praestekaer & Wien, 2008; Sevenans, 2017; Walgrave & Van Aelst, 2006; Yanovitzky, 2002). Accordingly, “politicians tend to adopt media issues, if not by solving the issue with real measures then, at least, by showing their commitment and displaying their responsiveness” (Walgrave & Van Aelst, 2006, pp. 100–101). Symbolic responses are “mostly rhetorical and do not have direct policy consequences” (Vesa et al., 2015, p. 282). Substantive actions, on the other hand, are more rare political responses involving policy and legislative changes (Vesa et al., 2015, p. 282).

Extending scholarly inquiry from the existence of a media agenda-setting effect on political activity to understanding *how* the news media exert this influence, scholars have identified a relationship between media framing and political responses to issues (Sevenans & Vliegenthart, 2016; Van der Pas, 2014; Walgrave et al., 2018). This is an area of research that is currently underrepresented in scholarly framing and agenda-setting literature, despite Van Aelst and Walgrave's (2011) recommendation for further studies interrogating the relationship between media framing and political agenda-setting. Consequently, this study aims to contribute towards filling this gap in research by exploring how frame building occurs episodically or thematically in reporting of VAW, and the influence on and of political agendas on these outcomes.

## Method

Examination of Australian news media framing of VAW and DV, and the associated implications for public and political responses to the issues, requires a framework for understanding how such frames are constructed in news reports. This study analyses the interplay between two framing junctures in the diachronic framing process model—news frames and public opinion indicators (Entman et al., 2009, p. 178) in the context of three cases of female murder. Content analysis is used to identify recurrent themes in the text (Hesmondhalgh, 2006) to understand how repetitive frames influence Australian public and political attitudes toward VAW and DV.

### News Media Article Collection

Initially, a search was conducted using the NewsBank database for relevant articles about each of the three murders analyzed. Articles were collected from Australia's major capital city publications: *The Advertiser, The Age, Courier Mail, Daily Telegraph, Herald Sun, Mercury, Sydney Morning Herald,* and *West Australian*, as well as the national newspaper *The Australian*. Data collection was limited to these publications, as each newspaper serves a large Australian capital city; thus, the publications were considered particularly powerful agenda setters and influential toward public opinion.

Specific search terms were used to locate reports of each incident in the NewsBank database within a 7-week period following each incident. To locate stories about Eurydice's murder, a search was conducted for original articles published between 15 June and 3 August 2018 using the terms “Princes Park” AND “Body,” and “Eurydice Dixon.” As Eurydice's murder was initially reported as an unidentified body found in Princes Park in Melbourne, the terms “Princes Park” and “Body” were included to capture reports that were published prior to confirmation of her identity. Larissa's murder was also initially reported as an anonymous “body in a barrel” prior to her identification. Thus, reports about Larissa's death published between 28 June and 16 August 2018 were obtained using the search terms “Body in barrel” OR “Larissa Beilby.” Conversely, Qi's murder was initially reported as a missing person case. As her identity was disclosed in all reports about her disappearance and death, reports published between 10 June and 29 July were obtained using the search term “Qi Yu.” In total, 108 original articles were obtained about Eurydice's murder, 30 about Larissa, and 11 about Qi.

### Analysis of News Articles

Following data collection, all articles were read to form a preliminary understanding of the news frames used to report each incident. A content analysis was then undertaken using NVivo 12 data management software. The articles were uploaded to NVivo and initially subjected to an auto-coding process whereby the NVivo software automatically identified predominant themes or codes in the texts. From this, the automated codes were manually applied by the researcher to analyze each article.

### News Media Framing Analysis

Articles were also manually analyzed for prevalent frames identified by previous studies of news discourses used to report VAW and DV including episodic (Berns, 1999; Bullock & Cubert, 2002; Hawley et al., 2017; Sellers et al., 2014) or thematic framing (Iyengar, 1990), and victim-blaming through framing the victims as “troubled” (Bullock & Cubert, 2002). A second coding process subsequently employed additional frames identified during the preliminary reading of all news reports, such as debate about VAW in Australia; public grieving; depicting the incident as a law and order issue; and framing of the offenders. Although most articles contained multiple frames, in the final stage of content analysis, each article was analyzed to identify the frame that featured most prominently.

These predominant frames were then categorized into thematic or episodic frames. Thematic frames were categorized as frames that referred to the incident in terms of a broader societal or cultural issue, or which referenced other similar incidents (Iyengar, 1990). In contrast, episodic frames were categorized as those which were specific to the incident itself, and/or attributed individual blame for the incident (Iyengar, 1990).

### Political Agenda-Setting Analysis

One of the challenges facing political agenda-setting studies is that it is difficult to conclusively prove a link between media coverage and political responses without access to the behind-the-scenes workings of political activity (Walgrave & Van Aelst, 2006; Wood & Peake, 1998). In consideration of these difficulties, this study adopts Van der Pas (2014) approach by taking statements (or lack thereof) from politicians in parliament as an indication of their political agenda. Consequently, Hansard records of the state parliaments of Victoria, Queensland and NSW where the crimes took place, as well as the Parliament of Australia, were searched for a 7-week period following each incident to quantify any references to the women made in parliament by politicians. Search terms included “Eurydice Dixon,” “Larissa Beilby,” and “Qi Yu.”

In addition, this study employs the approach of several other political agenda-setting studies in assuming that public statements from political figures which are reported in the media also reflect their agenda (Eshbaugh-Soha & Peake, 2005; Wood, 2007; Wood & Peake, 1998). Thus, political agenda-setting effects are measured in this study by analyzing politicians’ statements reported in the sample of news reports related to the incidents analyzed in this study over the 7-week period, as well as in Hansard parliamentary records during the same period. Agenda-setting effects assessed in this study include symbolic responses, where political figures show commitment and responsiveness to the issue (Walgrave & Van Aelst, 2006, pp. 100–101), as well as policy and legislative changes (Vesa et al., 2015, p. 282). The same frames that were identified in news content were analyzed in the context of news coverage of politicians' statements and Hansard’s statements.

## Findings and Discussion

Analysis of the three cases of VAW is applied to Entman et al.'s (2009) diachronic frame process model to explain how the media's framing of Eurydice Dixon's murder spurred public and political responses, which in turn led to greater and longer media attention. The media's reporting of Qi Yu and Larissa Beilby's murders, representing cases of DV, contrastingly were found not to produce public and political responses, nor sustained media attention. The reason for the different political agenda-setting consequences is proposed to stem from the thematic news framing of Eurydice's murder, and the episodic framing of the murders of Qi and Larissa.

### Media Framing Analysis: Thematic Versus Episodic Frames

**Figure 1.**
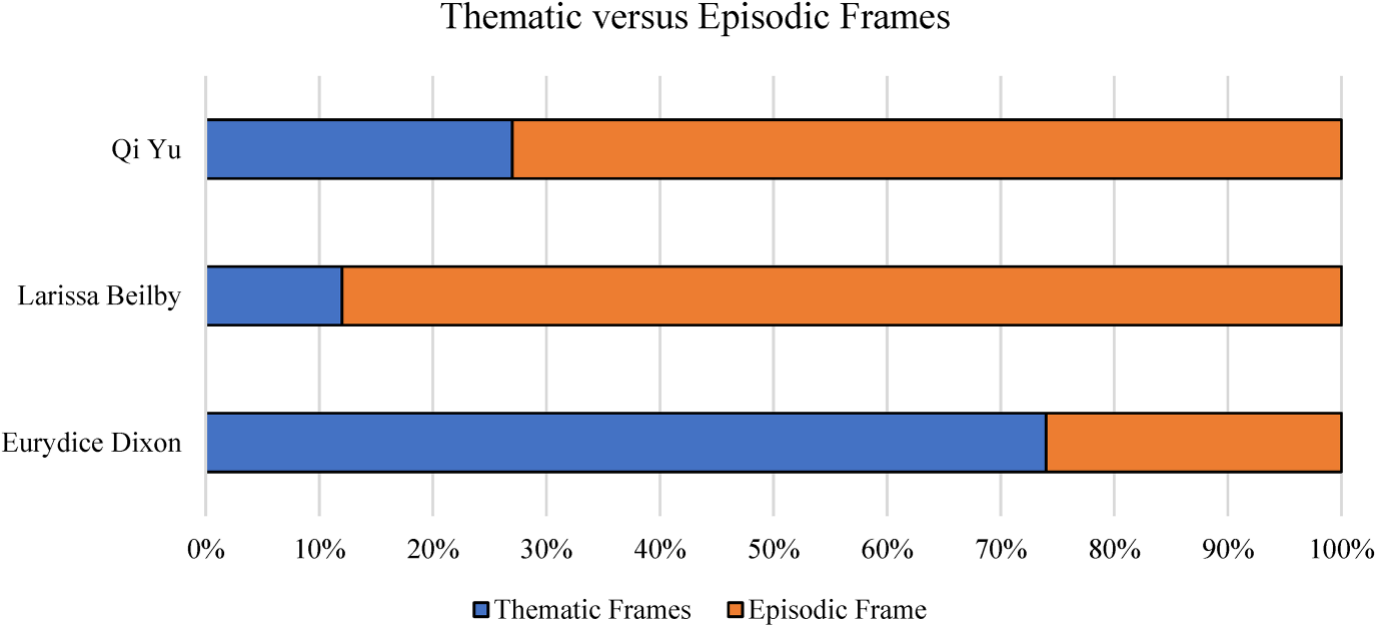
Percentage of thematic versus episodic frames used in news reporting. Eurydice Dixon, *n* = 107 articles; Larissa Beilby, *n* = 30 articles; and Qi Yu, *n* = 11 articles.

Eurydice's death was more extensively covered by the media, with 107 articles as compared to 30 articles about Larissa Beilby and 11 about Qi Yu. A comparison of the three cases of VAW (Figure 1) showed the case where the victim was a stranger to the offender, the murder of Eurydice Dixon, was framed by the media thematically (74%; Iyengar, 1990). Conversely, the two cases where the victims had domestic relationships with the offender, the murders of Qi Yu and Larissa Beilby, were framed episodically (73% and 88%; Iyengar, 1990). This finding adheres to other studies which find cases of DV are most often framed episodically (Easteal et al., 2019; Hawley et al., 2017; Sellers et al., 2014).

**Figure 2.**
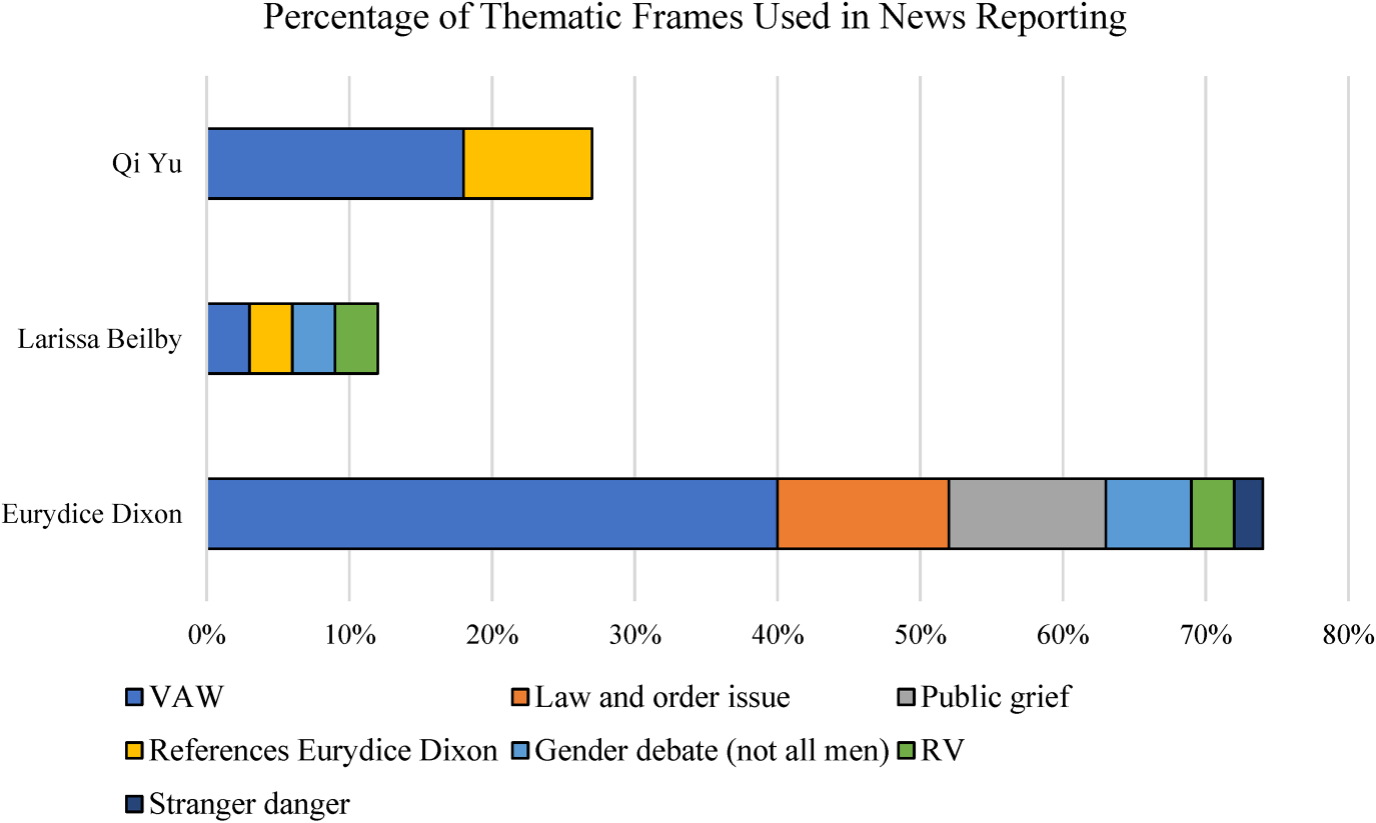
Percentage of thematic frames used in news reporting. Eurydice Dixon, *n* = 107 articles; Larissa Beilby, *n* = 30 articles; and Qi Yu, *n* = 11 articles.

Analysis of the specific frames used also reveals that thematic frames drew on dominant discourses about societal responsibility for crimes committed against these women, whereas episodic frames reflected discourses about individual and personal responsibility of the victims. Figure 2 depicts thematic frames used to report the three murders. Seventy-four percent of the frames used in reporting Eurydice's murder were thematic, with the predominant frame (40%) exemplifying VAW as a broader societal and cultural problem. These frames positioned Eurydice as an unfortunate victim of violence who was “unfairly taken” (Carlyon & Cahill, 2018), and who was not at “fault” (Credlin, 2018) for her murder. In many of these reports, Eurydice was framed as an “innocent victim,” demonstrating that some of the journalists reporting her murder saw her as a “good victim,” which may have contributed to the broader thematic framing of her death (Morgan & Simons, 2018).

Law and order (12%) and public grief (11%) were also significant thematic frames in reports of Eurydice's murder and were often referenced in articles alongside a VAW frame. Hundreds of people laid flowers at the site of Eurydice's murder in the days after her death, in a scene that was reminiscent of other high-profile national tragedies, such as the deliberate running down of pedestrians in Melbourne's Bourke Street in 2017, or the 2014 Lindt café siege in Sydney's CBD. Public grief after Eurydice's murder resembled a “collective catharsis” (Mikola et al., 2016, p. 336), whereby members of the public gather to collectively mourn the deaths of strangers after particularly tragic and high-profile incidents.

**Figure 3.**
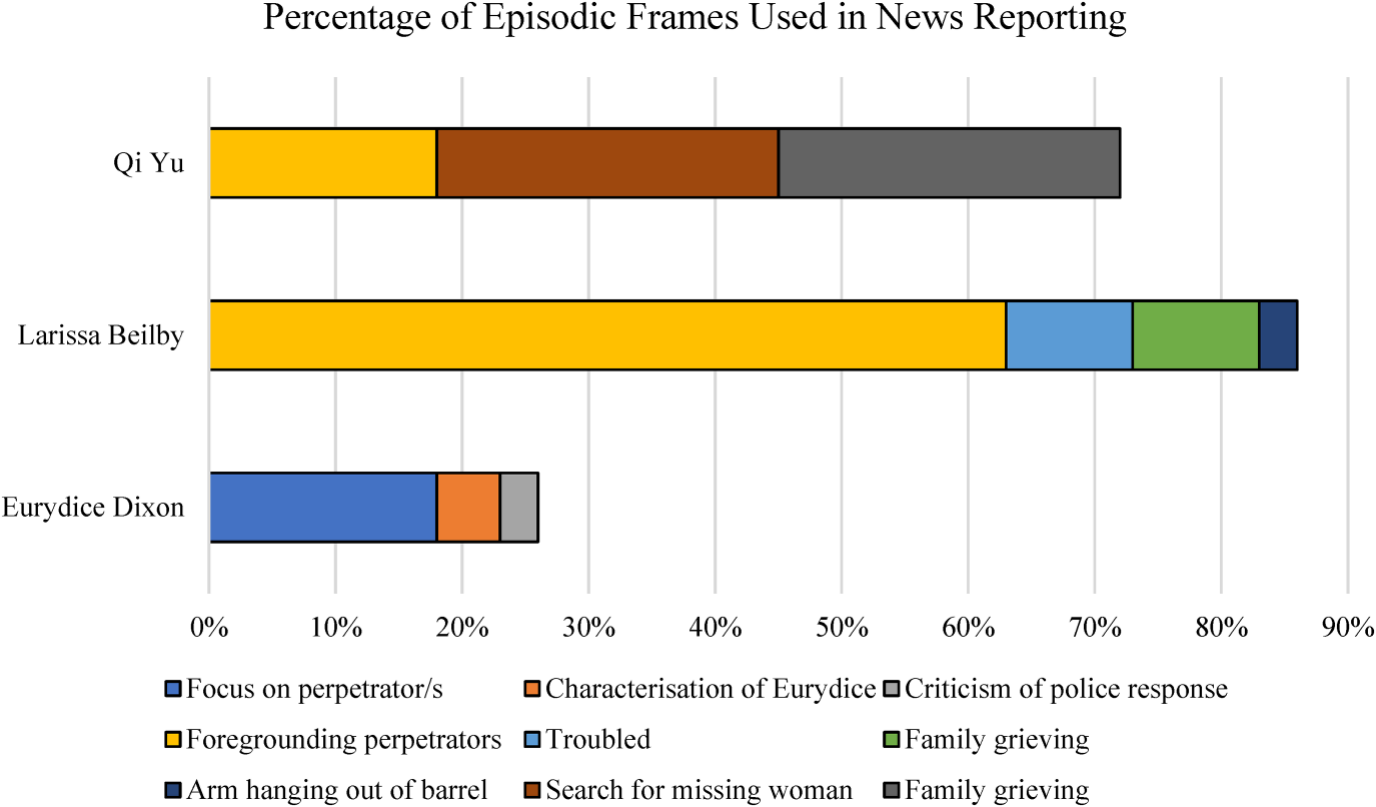
Percentage of episodic frames used in news reporting. Eurydice Dixon, *n* = 107 articles; Larissa Beilby, *n* = 30 articles; and Qi Yu; *n* = 11 articles.

The episodic frames used to report the murders of the three women are shown in Figure 3. Larissa's murder was predominantly framed as an individual crime committed by a violent and dangerous offender, with 63% of news reports about her death focused on the actions of the perpetrator. By foregrounding the actions of Larissa's murderer in news reports, the media framed him as a “dangerous individual,” rather than situating the incident as part of a broader societal problem of DV or VAW. Depiction of the perpetrators as “abnormal” is a common frame used to report DV (Bullock & Cubert, 2002; Worthington, 2013). Such framing individualizes the problem of DV and reduces expectations for politicians to take action.

The second-most prominent frame in reports of Larissa's murder, as shown in Figure 3, was an episodic victim-blaming frame which cast her as a “troubled” young woman who “fled into the arms of a violent man” (10%). For instance, one headline proclaimed: “Teen fled danger into the arms of death” (Kyriacou et al., 2018). This headline, in which she was depicted as “fleeing” into her killer's arms, positions Larissa as contributing to her death, a finding that aligns with other studies of reporting victims of DV (Berns, 1999; Saroca, 2013). This framing implies the victim “should have known better than to be involved with [her] abuser” (Fairbairn & Dawson, 2013, p. 152). By framing Larissa as “troubled,” news reports applied a victim-blaming frame of emotional turmoil, in line with the framing of DV victims as “abnormal” and thus somehow partially responsible for the violence perpetrated against them (Bullock & Cubert, 2002; Fairbairn & Dawson, 2013; Meyers, 1997).

Likewise, the predominant frame in reports of Qi's murder was that she was a missing person (27%), which also situated her death as an episodic crime, instead of reflecting a broader societal problem of VAW. In Qi's case, while no direct victim-blaming frames were identified, the absence of VAW and societal responsibility framing, in combination with the episodic framing of her death as an individual incident, served to indirectly frame her as somewhat responsible for the crime committed against her. Reports suggested that she was a “very nice girl” (Partridge, 2018), who had been killed by her flatmate in a dispute over money; thus, framing her as making the unfortunate decision to live with and have a dispute with a violent individual.

Absent from reporting about Larissa and Qi's death is any focus on law and order, or public grief. While the site of Eurydice's murder became a public memorial in the days after her death, public grieving and statements from high-profile figures did not feature in news reports about Larissa and Qi's deaths. Instead, as demonstrated in Figure 3, articles about Larissa and Qi focused on the private grief of their families and friends.

### Political Agenda-Setting: Political Actions and Public Grief About VAW

The media's thematic framing of Eurydice's murder, coupled with arguments from several commentators that the murder reflected a broader law and order problem in Melbourne (Harvey, 2018; Herald Sun, 2018a; Ross, 2018; Silvester, 2018) is proposed to have compelled the public and politicians to respond to VAW as “an affront to society” (Meyers, 1997, p. 53). The episodic framing of Larissa and Qi's murders, however, did not have the same effect.

**Figure 4.**
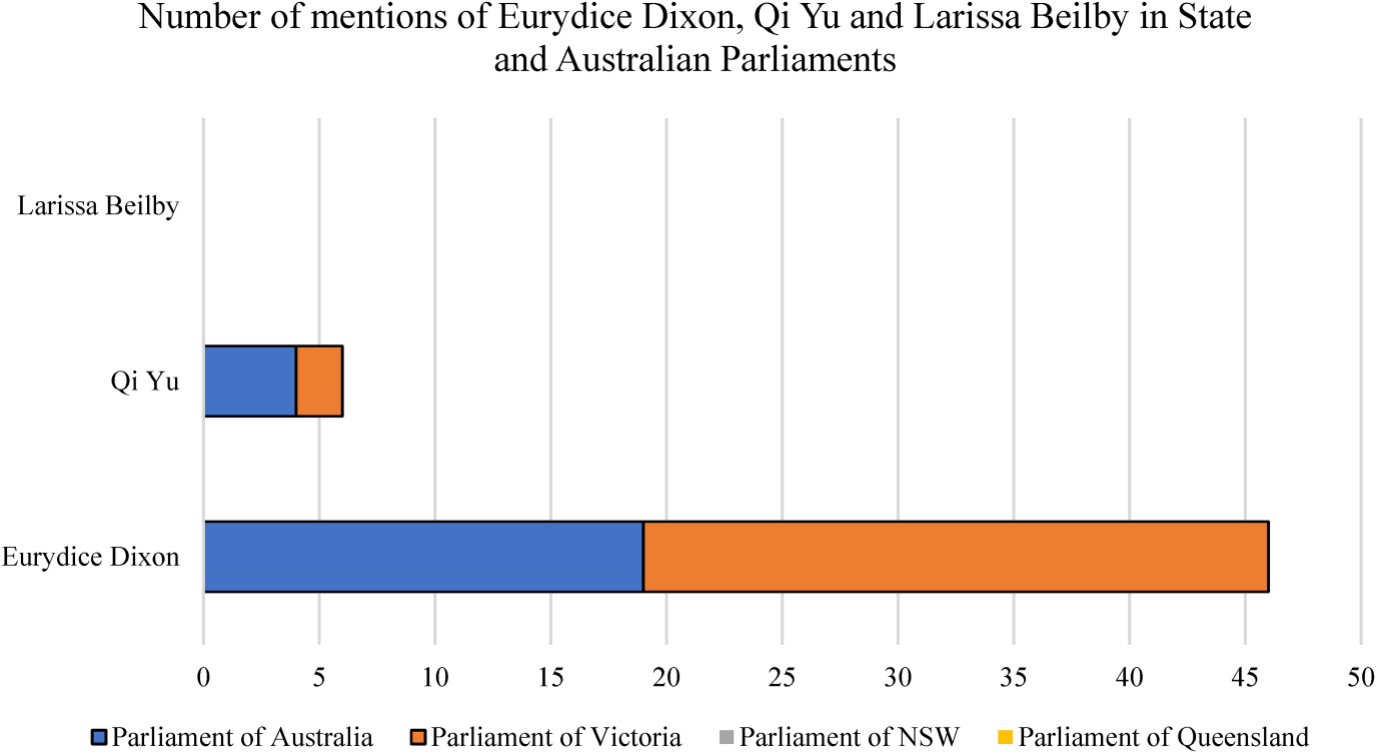
Number of times during data collection period that politicians mentioned each woman's name or referred to their death in Parliament.

As per Figure 2, 11% of thematic news frames about Eurydice's murder included reports of collective public grief and memorialization. This public response is theorized to have resulted from thematic media coverage which implied that the people of Melbourne should collectively grieve for Eurydice since her murder was represented as a societal problem. Furthermore, politicians also responded by linking the crime thematically to VAW. As depicted in Figure 4, during the 7 weeks after the murder, Eurydice was mentioned 27 times in the Parliament of Victoria. Such statements included condolence motions, a discussion of a broader cultural problem of DV and VAW, as well as to debate of the Justice Legislation Amendment (Family Violence Protection and Other Matters) Bill. Eurydice's death was also mentioned 19 times in the Parliament of Australia during the data collection period—14 of which were statements by Members or Private Members’ Businesses regarding the Prevention of Violence Against Women. This finding is evidenced by statements from several prominent political figures, including the Premier of Victoria and the Australian Prime Minister, that Eurydice's death represented a social and cultural problem of VAW in Australia.

These findings suggest that the thematic media reporting of Eurydice's death had a political agenda-setting effect. Where other studies have suggested media attention leads to political attention (Van der Pas et al., 2017; Vliegenthart et al., 2016; Walgrave & Van Aelst, 2016), this study suggests thematic VAW frames have a greater influence on political and public action than episodic frames.

Political agenda setting, however, was predominantly limited to public statements by politicians, rather than identifiable and meaningful political action. It should be noted, as per Elmelund-Praestekaer and Wien (2008), that political action such as policy change, on an issue as systemic as VAW, is likely to take longer than the 7 weeks of analysis in this study. Additionally, although no immediate policy action resulted from the Victorian state parliament discussion of VAW in relation to Eurydice's death during the analysis period, the Victorian government at this time were already responding to the findings of the 2015 Royal Commission into Family Violence. In January 2023, the Victorian government announced they were implementing policies in response to the last of the 227 recommendations from this Royal Commission, showing a commitment to long-term reform (Department of Families, Fairness and Housing, 2023).

Several politicians also advocated to improve women's safety following Eurydice's death. For instance, in a Facebook post 2 days after Eurydice's murder, Premier Andrews emphasized his government's commitment to ending VAW, and in particular DV:

… Eurydice did not make it home safe. In a few days, women across Melbourne will gather in Princes Park for a vigil of her life. And they will do so firm in the knowledge that Eurydice died because of her attacker's decisions – not because of her own. They’re right. And we need to accept that fact, too. We’ll never change a thing until we do. We’ll never change this culture of violence against women. All women. We’ll never change the fact that one woman in this country dies every week at the hands of a partner or former partner – someone they loved, in the safety of their own home. We’ll keep asking “Why didn’t she leave him?” instead of asking “Why did he hurt her?” We’ll keep asking “Why was she alone in the dark?” instead of asking “Why was he?” (Andrews, 2018)

Similarly, Prime Minister Turnbull advocated that women “must be safe everywhere,” and that: “We must change the hearts of men to respect women. We start with the youngest … the little boys, our sons and grandsons, and make sure that they respect … all the women in their lives” (cited in Allison, 2018).

There is also continued evidence the Victorian government is following through on their commitment to enact substantive measures following Eurydice's death to address VAW, including particularly DV. This political agenda was emphasized in the wake of Eurydice's murder by Victorian Premier Andrews’ statement that his government has: “a comprehensive plan that leads our nation in its scale and innovation to change attitudes and, therefore, behavior [towards VAW]” (Argoon et al., 2018). The plan includes the establishment of a new agency in Victoria aimed at tackling DV and family violence (Argoon et al., 2018), and a rolling action plan to combat family violence, based on the recommendations of the Royal Commission. More recently, the Victorian government Budget 2022/23 invested $240 million in initiatives directly responding to DV, such as expanding “critical refuge and crisis accommodation for victim-survivors who cannot remain safely at home” and $43 million for family violence services (Williams, 2022).

There was furthermore some evidence of other actions taken by politicians and institutions after Eurydice's murder. This action, however, tended to respond to VAW as an episodic law and order issue, rather than a systemic problem of DV. For example, police announced 24-hr police patrols in the park where Eurydice was killed (Fox Koob & Pearson, 2018). Addressing community safety concerns, Melbourne Lord Mayor Sally Capp stated that “I believe that we are also doing everything we can to make [Melbourne] safer” (cited in Cunningham & Pearson, 2018). Further joint initiatives between politicians and the authorities included an announcement that “The City of Melbourne, Victoria Police and the state government will examine lighting, CCTV and whether Protective Services Officers could be redeployed to crime hot spots” (Herald Sun, 2018b).

The same agenda-setting response from the public, politicians, and the authorities was almost entirely absent after the deaths of Larissa and Qi. Only one reference to safety was made in articles about Qi, which was by Jenna Price, a Fairfax columnist and academic who researches VAW. In the article, Jenna also noted the lack of media attention and public mourning for Qi and other women murdered by men known to them: “For these women, we held no nationwide vigils. If there was grief, it was restricted to family and friends only” (Price, 2018). Likewise, the only references to safety in Larissa's case were articles stating that she was reported missing on 15 June and that police held fears for her safety (Chamberlin et al., 2018; Kyriacou et al., 2018). No criminal, legislative, or safety measures for victims of DV were proposed by the media, politicians, or authorities in response to Qi or Larissa's murder.

This absence reflects media and political attitudes that crimes committed in public, like Eurydice's murder, are a threat to society (Carter, 1998; Meyers, 1997). Conversely, those committed in the home are framed as a private or individual matter, and therefore as representing less of a threat to the community than crimes perpetrated by strangers (Hawley et al., 2017). The analysis found 13 articles about Eurydice asserting that she and other women have the right to feel safe while walking home at night. Yet, no articles mentioned Larissa and Qi's right to be safe in their own home. Episodic, individualized framing is found to lower public and media expectations of political response and action toward an issue, as these frames “displace attention away from larger social conditions” by focusing problems on individuals instead of society (Kim et al., 2010, p. 577).

### Applying the Diachronic Framing Process Model

**Figure 5.**
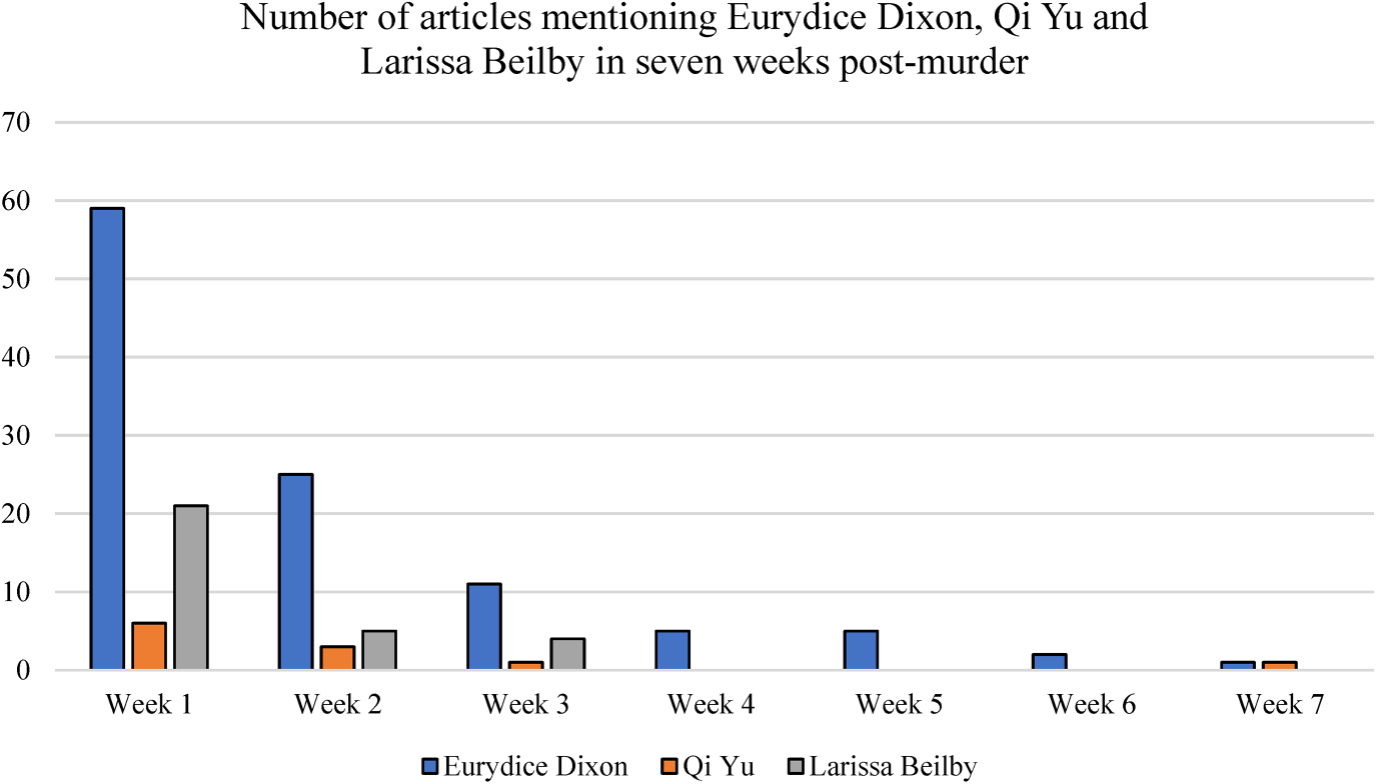
Total number of news articles each week across the 7-week data collection period.

Entman et al.'s (2009) diachronic framing process model can be applied to understand why thematic framing influenced political actions and statements about VAW, which in turn led to more and prolonged media attention on Eurydice's murder, but episodic framing did not (Figure 5). By applying this model to the reporting of VAW, this study also helps to clarify what Entman et al. (2009, p. 188) refer to as the flow of framing and power between media, elite politicians, and the public and how this interaction influences public discourse and political agenda setting.

The diachronic framing process model theorizes that dominant frames reported by the media will influence public opinion indicators (Entman et al., 2009). In the case of Eurydice's murder, thematic reporting (Figures 1 and 2) is proposed to have elicited nonstrategic communication in the form of an outpouring of grief by the public, and strategic communication by politicians in the form of public statements and pledges to address the societal problem of VAW, including DV.

The model also suggests that these public opinion indicators can reciprocally act as feedback loops to influence news media framing to again give the issue attention (Entman et al., 2009). Similar to Shah's (2019) finding that social media outrage and activism about the 2012 Delhi gang rape led the media to shift reporting from episodic frames to thematic frames, public grief about Eurydice's murder is also proposed to have influenced the media's ongoing thematic framing of the crime. As per Figure 5, public opinion frames encompassing public grief, as well as political statements and actions, are proposed to have generated further media coverage and sustained the news coverage of the incident over a long period.

In contrast, news coverage of Larissa and Qi's deaths was framed episodically with a clear beginning and end when each ‘episode” reached a natural conclusion in the eyes of journalists. In the absence of thematic frames to sustain news coverage, and political action and attention, Figure 5 shows that after Larissa's funeral in Week 3, and when it became evident in Week 3 that Qi's body may never be found, news reporting of both cases came to a close.

**Figure 6.**
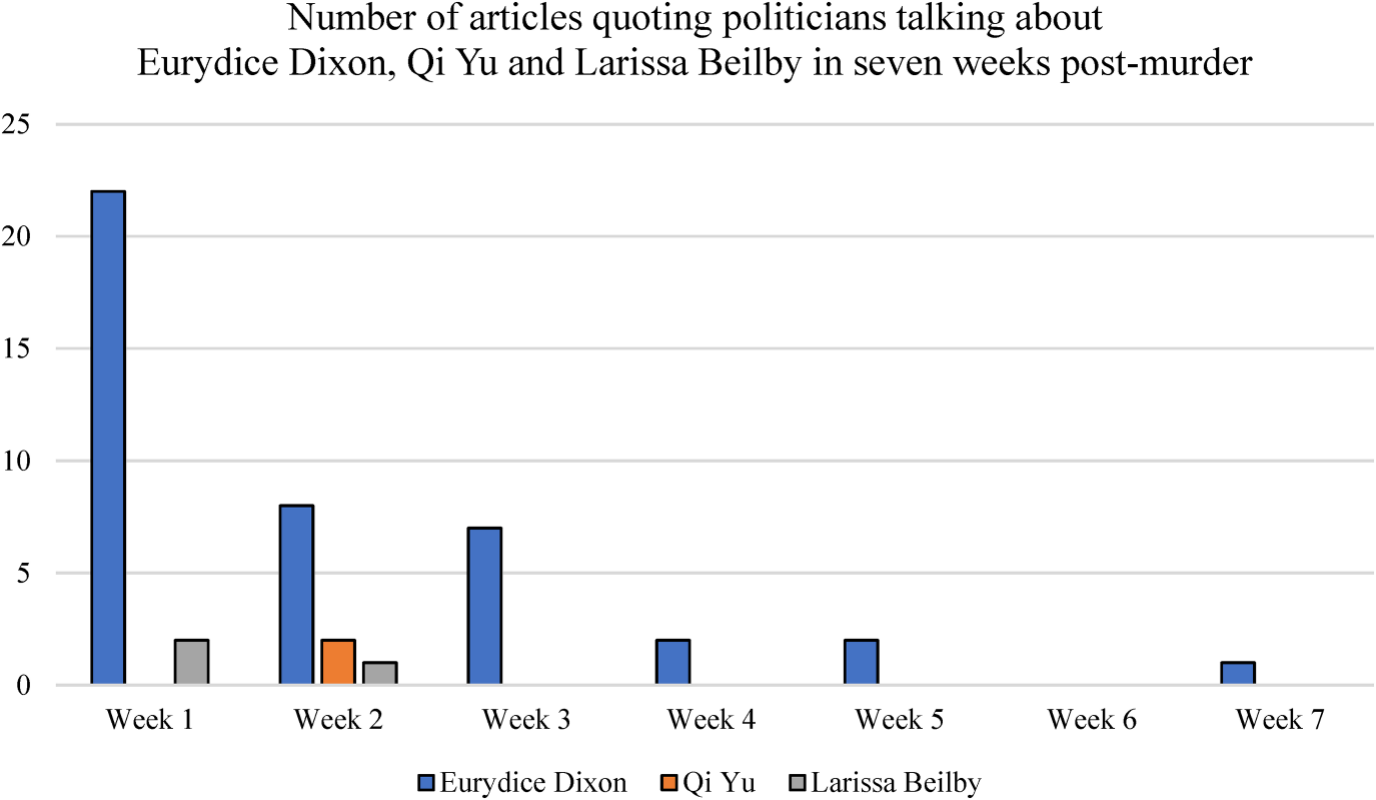
Total number of news articles each week mentioning politicians across the 7-week data collection period.

Political statements and actions in response to Eurydice's murder were also found to influence sustained media attention on this crime. Figure 6 shows that in the first week after Eurydice's death, more than one-third of articles mentioned politicians, and that politicians continued to be mentioned in stories about her in six of the seven weeks of reporting analyzed. As discussed, responses from political figures varied, but predominantly involved symbolic statements rather than meaningful, substantive action, as is often the case with issues that arise to the attention of politicians from being highly salient on the media agenda (Elmelund-Praestekaer & Wien, 2008; Sevenans, 2017; Walgrave & Van Aelst, 2006; Yanovitzky, 2002). It is noteworthy that despite Eurydice's murder being unrelated to DV, many of the statements made by politicians following Eurydice's death referred to the problem of DV in Australia, as well as VAW more broadly.

Further sustaining the media attention to Eurydice's death was an incident in the third week of reporting, when federal politician David Leyonhjelm was accused of “slut-shaming” another federal politician, Sarah Hanson-Young, through remarks he made in the Parliament of Australia. These remarks were immediately picked up and framed as “harmful” by both journalists and politicians, several of whom referenced Eurydice's murder in saying that Senator Leyonhjelm's remarks were indicative of a broader culture of misogyny in Australia; thus, further building on the primary thematic frame and political agenda that had dominated public discussion of Eurydice's murder.

While many political statements were made and reported in relation to the death of Eurydice, who was killed by a stranger, as demonstrated in Figure 4, statements from political figures about two actual victims of DV—Larissa and Qi—were minimal or nonexistent. Qi was mentioned 4 times in Australian Parliament statements by Members or Private Members’ business regarding the prevention of VAW, and twice in statements about Eurydice's death in the Parliament of Victoria. In stark contrast, Larissa Beilby was not mentioned in any statements by politicians in the Parliaments of Australia, Victoria, or her home state of Queensland.

Other research offers insights into the application of the diachronic frame process model (Entman et al., 2009) to help explain the reasons why thematic framing of VAW influences political agendas, which in turn increases the longevity of news stories, where episodic frames do not. Sevenans (2017) argues that politicians use “heightened media attention” as a motivation to take political action, even when they already knew about the societal issue before the news gave attention to it. Likewise, Green-Pedersen and Stubager (2010) contend that politicians are more likely to respond to issues in the news that they consider to be politically advantageous in some way.

Whether politicians are motivated to take political action by the media attention afforded to an issue, or because they view the issue as being politically advantageous, both perceptions provide an explanation for the dichotomy between the political attention paid to Eurydice's murder, in contrast to the absence of political attention following the deaths of Qi and Larissa. At the time of Eurydice's death, the presence of a cultural problem of VAW had already been salient on the media agenda and thus known to politicians for some time, having been raised following high-profile murders of other women such as Jill Meagher, and the international #MeToo movement. Conversely, although DV is a long-standing issue, the lack of significant media attention given to the problem means that politicians have not had to formulate a response to the issue, as they are unlikely to be expected by the media and public to spontaneously respond to such incidents.

Politicians, and male politicians in particular, may also view taking a publicly progressive stand against the cultural problem of VAW as politically expedient, especially in the wake of broader cultural debate about VAW prompted by the #MeToo movement. As Walgrave and Van Aelst (2006, p. 101) contend: “The quicker their reaction, the higher the chance they can get the line of the day out, get their face on TV, and connect with the public. Instant issue adoption maximizes media exposure.”

The Victorian government's previous commitment to address family violence, and by extension VAW, may also explain why Premier Andrews provided a thematic response to Eurydice's murder, unlike the premiers of the states where Larissa and Qi were murdered. As pointed out by Berns (2017), it is easier for the media to episodically frame VAW as a crime or medical issue, because the social problem of VAW is much harder to tackle. The same idea applies to politicians. Since the Victorian government's response to the 2015 Royal Commission into Family Violence was already being implemented, this enabled Premier Andrews to tie one episode of VAW—albeit an incidence of stranger violence—thematically to an existing policy agenda. This idea aligns with Van der Pas’s (2014) suggestion that political parties are more likely to respond to media attention about an issue when media frames align with the party's frames. Andrews was well-equipped to respond to Eurydice's murder, given that it aligned with his government's progressive agenda of eliminating family violence and VAW. Thus, it is conceivable that had Larissa or Qi been murdered in Victoria, their deaths may have elicited more media and political attention than they received, as the Victorian government had already introduced significant policies and reforms aimed at reducing and eliminating family violence in Victoria.

Overall, the political response to Eurydice's murder was what Walgrave and Van Aelst (2006, p. 101) have termed a “fast symbolic reaction”—a common response from politicians to salient issues on the media agenda. In contrast, as this study has shown, the political response to Larissa and Qi's deaths was that of “no reaction” (Walgrave & Van Aelst, 2006, p. 101).

## Conclusions

The news framing of the three murdered women varied significantly. Eurydice was framed as an innocent victim of VAW who was “unfairly taken” (Carlyon & Cahill, 2018), while Qi and Larissa were framed as partially contributing to the circumstances of their deaths. Furthermore, Larissa was framed as a “troubled” teenager who “fled into the arms of a violent man,” while Qi was framed as a “nice girl” who was killed by her flatmate over money (Partridge, 2018). For these reasons, Eurydice's murder generated substantially more media attention than Larissa or Qi's, which in turn created a public and political agenda-setting effect.

Paradoxically, although Eurydice was killed by a stranger, her death was linked to DV by politicians, particularly the Victorian government who already had in place an agenda to address VAW through measures focused on the most common form of VAW—DV. Public attention and grieving, prompted by the extensive media coverage of the incident, compelled politicians to publicly respond and propose “solutions” following her attack. Such responses from politicians were absent after Qi and Larissa's murders.

Entman et al.'s (2009) diachronic frame process model helps to explain why thematically framed news reports that present VAW as a societal responsibility are more likely to lead to political focus on an issue. This is seen through the heightened attention on Eurydice's murder by politicians, which also generated more media coverage. Public outpourings of grief were also influential on continued media attention. Thematic frames thus influence the news media to give more sustained and regular coverage to VAW. Episodic framing, on the other hand, facilitates inaction as political and legislative responses are not demanded or expected by the media and the public when issues are framed in this way. Although immediate political responses to both issues are frequently symbolic rather than substantive, thematic framing can help policymakers reinforce the importance of an existing agenda and increase public support for reform efforts. The lack of policy responses to incidents of DV, conversely, perpetuates long-standing inaction toward the issue and stifles further momentum to address the issue, either symbolically or substantively.

Framing influences public and social attitudes toward important issues and therefore plays a significant role in establishing and swaying public expectations of politicians’ responses to issues. As per Sutherland et al.'s (2016, p. 34) argument that media reporting of VAW reflects society's “confusion and ambivalence” about a problem at crisis levels in Australia, this research demonstrates it is no wonder that the public is confused when VAW “stranger danger” incidents prompt thematic framing by the media, politicians and the public, where much more common incidents of DV do not. This research suggests that future media framing of DV incidents should focus on framing such incidents thematically—as indicative of a widespread cultural problem of VAW. In line with Easteal et al.'s (2022) study which showed positive outcomes from journalist training in VAW reporting, journalists should be taught that thematic framing is likely to lead to more political action to address the issue, whereas episodic framing is stigmatizing and implies the victim is at fault. This research suggests that improving thematic reporting would increase public understanding and pressure on politicians to act, and may provide the impetus for substantive long-term measures such as those adopted by the Victorian government to prevent DV and support victims to leave dangerous situations. Responsibility for improving public understanding of VAW as a societal rather than individual problem also belongs to politicians, with this study showing when they give thematic attention to incidents of VAW, they help sustain the issue on the media agenda. Furthermore, greater emphasis is required in news reports and political communication about the “right” of women to feel safe in their own homes, in the same way the media and politicians emphasized Eurydice and other women's “right” to feel safe walking alone late at night.

These findings provide important insights into the interplay between the thematic framing of VAW and the political will to address this societal problem. One limitation of the study is that it is confined to analyzing news reports of the murders of only three women. Future research would benefit from a broader sample of cases of VAW and DV, both domestically and internationally, to further understand the relationship between media framing and political responses to these issues. Additionally, since policies addressing VAW can take time to be developed and implemented, a longer-term study would have the benefit of tracing longer-term reform to thematic discussion of VAW in the media to measure whether political agenda setting had a sustained societal impact.
